# Hsa-miR-21-3p associates with breast cancer patient survival and targets genes in tumor suppressive pathways

**DOI:** 10.1371/journal.pone.0260327

**Published:** 2021-11-19

**Authors:** Arsalan Amirfallah, Hildur Knutsdottir, Adalgeir Arason, Bylgja Hilmarsdottir, Oskar T. Johannsson, Bjarni A. Agnarsson, Rosa B. Barkardottir, Inga Reynisdottir

**Affiliations:** 1 Cell Biology Unit, Department of Pathology, Landspitali–The National University Hospital of Iceland, Reykjavik, Iceland; 2 Biomedical Center, Faculty of Medicine, University of Iceland, Reykjavik, Iceland; 3 Department of Biomedical Engineering, Johns Hopkins University, Baltimore, Maryland, United States of America; 4 Molecular Pathology Unit, Department of Pathology, Landspitali–The National University Hospital of Iceland, Reykjavik, Iceland; 5 Department of Pathology, Landspitali–The National University Hospital of Iceland, Reykjavik, Iceland; 6 Department of Oncology, Landspitali–The National University Hospital of Iceland, Reykjavik, Iceland; 7 Faculty of Medicine, University of Iceland, Reykjavik, Iceland; Manipal School of Life Sciences, Manipal Academy of Higher Education, INDIA

## Abstract

Breast cancer is the cancer most often diagnosed in women. MicroRNAs (MIRs) are short RNA molecules that bind mRNA resulting in their downregulation. MIR21 has been shown to be an oncomiR in most cancer types, including breast cancer. Most of the effects of miR-21 have been attributed to hsa-miR-21-5p that is transcribed from the leading strand of MIR21, but hsa-miR-21-3p (miR-21-3p), transcribed from the lagging strand, is much less studied. The aim of the study is to analyze whether expression of miR-21-3p is prognostic for breast cancer. MiR-21-3p association with survival, clinical and pathological characteristics was analyzed in a large breast cancer cohort and validated in three separate cohorts, including TCGA and METABRIC. Analytical tools were also used to infer miR-21-3p function and to identify potential target genes and functional pathways. The results showed that in the exploration cohort, high miR-21-3p levels associated with shorter survival and lymph node positivity. In the three validation cohorts, high miR-21-3p levels associated with pathological characteristics that predict worse prognosis. Specifically, in the largest validation cohort, METABRIC (n = 1174), high miR-21-3p levels associated with large tumors, a high grade, lymph node and HER2 positivity, and shorter breast-cancer-specific survival (HR = 1.38, CI 1.13–1.68). This association remained significant after adjusting for confounding factors. The genes with expression levels that correlated with miR-21-3p were enriched in particular pathways, including the epithelial-to-mesenchymal transition and proliferation. Among the most significantly downregulated targets were MAT2A and the tumor suppressive genes STARD13 and ZNF132. The results from this study emphasize that both 3p- and 5p-arms from a MIR warrant independent study. The data show that miR-21-3p overexpression in breast tumors is a marker of worse breast cancer progression and it affects genes in pathways that drive breast cancer by down-regulating tumor suppressor genes. The results suggest miR-21-3p as a potential biomarker.

## Introduction

MiRs are small non-coding RNAs that regulate gene expression by binding to 3´UTRs of target mRNAs to cause them to be unstable and/or degrade. MiRs are transcribed as long primary miRs (pri-miRs) and subsequently processed to much shorter pre-miRs that can give rise to two mature molecules miR-5p and/or miR-3p [1]. MiRs can be located both intra- and intergenically and can be transcribed independently from their own promoter or the promoter of the gene in which they reside [1].

MIR21 overlaps with the vacuole membrane protein 1 (VMP1), sharing about 1 kb of sequence [2]. The two genes have separate promoters, with pri-miR-21 transcribed from its own promoter, miPPR-21 [3, 4]. That promoter is a site of active transcription, as it contains binding sites for transcription factors such as STAT3, AP-1, C/EBP and p53 among others [5] (reviewed in [6]). Both VMP1 and MIR21 have their own polyadenylation sites, located upstream of the miR-21 hairpin for VMP1 but downstream of the hairpin for MIR21 [2, 7]. This suggests that the two genes are transcribed independent of each other.

MIR21 is well studied in cancer and is reported as a potential diagnostic, prognostic, and predictive biomarker in many cancer types, including breast cancer (reviewed in [6]). A meta-analysis of breast cancer demonstrated that elevated levels of miR-21 predict poor prognosis for breast cancer patients when miR-21 was measured in breast tumors and in tumor cells circulating in serum [8]. Most clinical studies that analyzed miR-21 (see studies within this meta-analysis [9]) used probes that measured miR-21 (miR-21-5p) but not miR-21* (miR-21-3p). Overexpression of miR-21 was reported to enhance cellular proliferation and induce invasion and metastasis. This was achieved through interaction with its target genes, many of which are known tumor suppressor genes, *e*.*g*., division cycle 25A (CDC25A), programmed cell death 4 (PDCD4), tropomyosin 1 (TPM1) and phosphatase and tensin homolog (PTEN) [10–13]. Only a small number of studies focused on the role of miR-21-3p in breast cancer survival, with conflicting results.

MiR-21-3p levels were reportedly higher in breast tumors than in normal breast tissue [14, 15]. In a clinical study performed using triple negative breast tumors from The Cancer Genome Atlas (TCGA), low miR-21-3p expression associated with shorter overall survival and, when combined in a panel with two other miRs, was suggested to be a prognostic marker predicting shorter survival [14]. In another study using breast tissue and serum samples from TCGA, high miR-21-3p expression was suggested to be a non-invasive prospective marker for detection of early-stage breast cancer (when it was used in a panel with two other miRs) [16]. Another study found that, when analyzing miR-21-3p paired with another miR, the pair could distinguish between breast tumor tissue and benign lesions [17]. Although miR-21-3p studies in breast cancer are few, emerging evidence suggests it plays a role in the disease. Thus, the focus of this study is miR-21-3p and its potential role as a biomarker in breast cancer. Here, we examined whether miR-21-3p expression was associated with tumor malignancy, shorter survival, and a poor prognosis for breast cancer patients. In addition, our bioinformatic analyses sought pathways and targets modulated by miR-21-3p.

Our results show that elevated miR-21-3p expression associated with clinical and pathological characteristics that indicate disease severity and shorter breast-cancer-specific survival. Bioinformatic analyses revealed that genes positively correlating with miR-21-3p expression were enriched in pathways that induce proliferation and the epithelial-to-mesenchymal transition, whereas genes that negatively correlated included candidates that when under expressed were implicated in tumor progression.

## Materials and methods

### Cohorts and clinical data

Breast cancer patients in cohort-1 (n = 158; diagnosed 1987 to 2003) and cohort-2 (n = 291; diagnosed 2003–2007), and the collection of relevant patient and tumor data have been reported [18, 19]. Primary fresh frozen tumors and normal breast tissue were obtained from the Department of Pathology. In cases of small tissue specimens, normal (non-neoplastic) breast tissue was dissected as far away from the tumor as possible and an adjacent tissue slice stained with hematoxylin and eosin (HE) to confirm absence of tumor cells in the normal sample. Informed consent was obtained from all subjects involved. The study was approved by the National Bioethics Committee of Iceland (VSN-15-138). Data for the breast cancer patients from TCGA cohort (Firehose Legacy, n = 1108) [20], diagnosed 1988–2013, and the METABRIC cohort (n = 2509) [21–23], diagnosed 1980–2005, were collected through the cBioPortal [24, 25]. To our knowledge, normal breast samples from METABRIC are not available.

### DNA and RNA isolation

DNA and total RNA were extracted from fresh frozen non-neoplastic breast tissue samples from cohort-2 using the Allprep kit DNA/RNA/miRNA (Qiagen no. 80224). Nucleic acids were not isolated from normal tissue samples from cohort-1 as only a handful of samples exist. Since normal breast tissue is enriched in fatty tissue and stromal cells, as compared to the tumor, a section of normal breast tissue was stained with eosin and hematoxylin, before extraction, to ensure the presence of normal epithelial breast cells in the specimen. Thirty-five samples were chosen for continuation based on the presence of normal breast tissue, RNA quantity and RIN (RNA integrity number) value. DNA and total RNA was extracted from cohort-2 by the same method used for normal breast tissue [18], but total RNA from cohort-1 was extracted with Trizol, as described [19].

### MiRNA and mRNA data

MiR-21 expression was quantified in breast tumors from cohorts-1 and 2. A miRCURY LNA RT Kit (Qiagen) was used to generate cDNA from 5 ng/μl of RNA from breast tumors and normal breast tissue from cohorts-1 and 2 by following the manufacturer’s protocol. Quantitative-PCR (qPCR) was performed using miRCURY LNA SYBR Green PCR Kit by following the manufacturer´s protocol (Qiagen). MiRCURY primer sets for hsa-miR-21-3p (YP00204302) and hsa-miR-16-5p (YP00205702) were used, with the latter as a reference gene. The reference gene, hsa-miR-16-5P, was chosen based on guidelines from the manufacturer of miRCURY kits, published studies [26, 27], and our testing miRs in cohort-1. Hsa-miR-16-5p showed little variability between the tumor samples when analyzed on its own or as a partner in a set with miR-29a-3p and thus was used as the sole reference gene. Reactions were performed in triplicate using 40 cycles according to the manufacturer’s protocol. Values for miR-21-3p and mRNAs from TCGA were retrieved through the cBioPortal [24, 25]. Two libraries, Illumina Genome Analyzer (238 patients) and Illumina HiSeq 2000 (717 patients), were used to generate the RNA-Seq data that are the basis for the miR-21-3p isomiR extraction [20]. Nine isomiRs representing miR-21-3p were used, based on their differential expression, higher in tumors than normal (S1 Fig and S1 Table). MiR-21-3p expression values from METABRIC were not available through the cBioPortal and so were retrieved from the European Genome-phenome Archive (EGA: https://ega-archive.org/) from study EGAS00000000122, dataset accession number EGAD00010000438. The probe used to detect miR-21-3p on the Agilent microarray is 15 nucleotides and binds the reference isomiR sequence, and possibly other isomiRs of miR-21-3p [28]. MiR-21-5p expression values were also retrieved from EGA. For the bioinformatic analyses, normalized mRNA values for the METABRIC cohort were retrieved from the same study (dataset accession EGAD00010000434).

### Statistical analyses

Patients that lacked data for mRNA, miRNA, or survival were excluded from the analyses. The number of patients in each cohort was 139, 281, 946, and 1174 for cohort-1, cohort-2, TCGA, and METABRIC, respectively. The miR-21-3p values from cohorts-1 and 2 and TCGA were transformed with log2 to normalize the data. The miR-21-3p and miR-21-5p values from METABRIC had been normalized [29]. All miR values were centered at 0. VMP1, TUBD1, RPS6KB1, BCAS3, and PPM1D mRNAs from METABRIC that were retrieved from the cBioPortal had been normalized (Z scores). The statistical program R version 3.5.3 was used for the analyses [30]. Correlation between miRNA and mRNA expression was calculated by Pearson’s product moment correlation using normalized values. For the bioinformatic analyses, Stouffer’s method for meta-analysis was used to combine results from TCGA and METABRIC, $Z=\frac{\sum_{i=1}^{k}Z_{i}}{\sqrt{k}}$, where Z are the Z-scores and k = 2 representing TCGA and METABRIC. Genes were considered significantly correlated using the 5% FDR (false discovery rate) threshold in each cohort and the more stringent 5% FWER (familywise error rate) threshold in the meta-analysis. The association of miR-21-3p with clinical and pathological characteristics was performed with Student’s t-test or ANOVA. Expression levels in breast tumors and normal breast tissue were compared with a paired t-test. Kaplan-Meier and log-rank tests were calculated to estimate survival using the survival and survminer packages in R. Tumors were classified into high- and low-expressing tumors, based on the median miR-21-3p expression values in each cohort. Cox regression analyses calculated hazard ratios (HR) and the effect of tumor characteristics with an independent effect on survival. Characteristics with numerical values were analyzed as both categorical and continuous variables. P-values below 0.05 were considered significant.

## Results

### MiR-21-3p associates with metastasis and shorter disease-free survival

**Fig 1:**
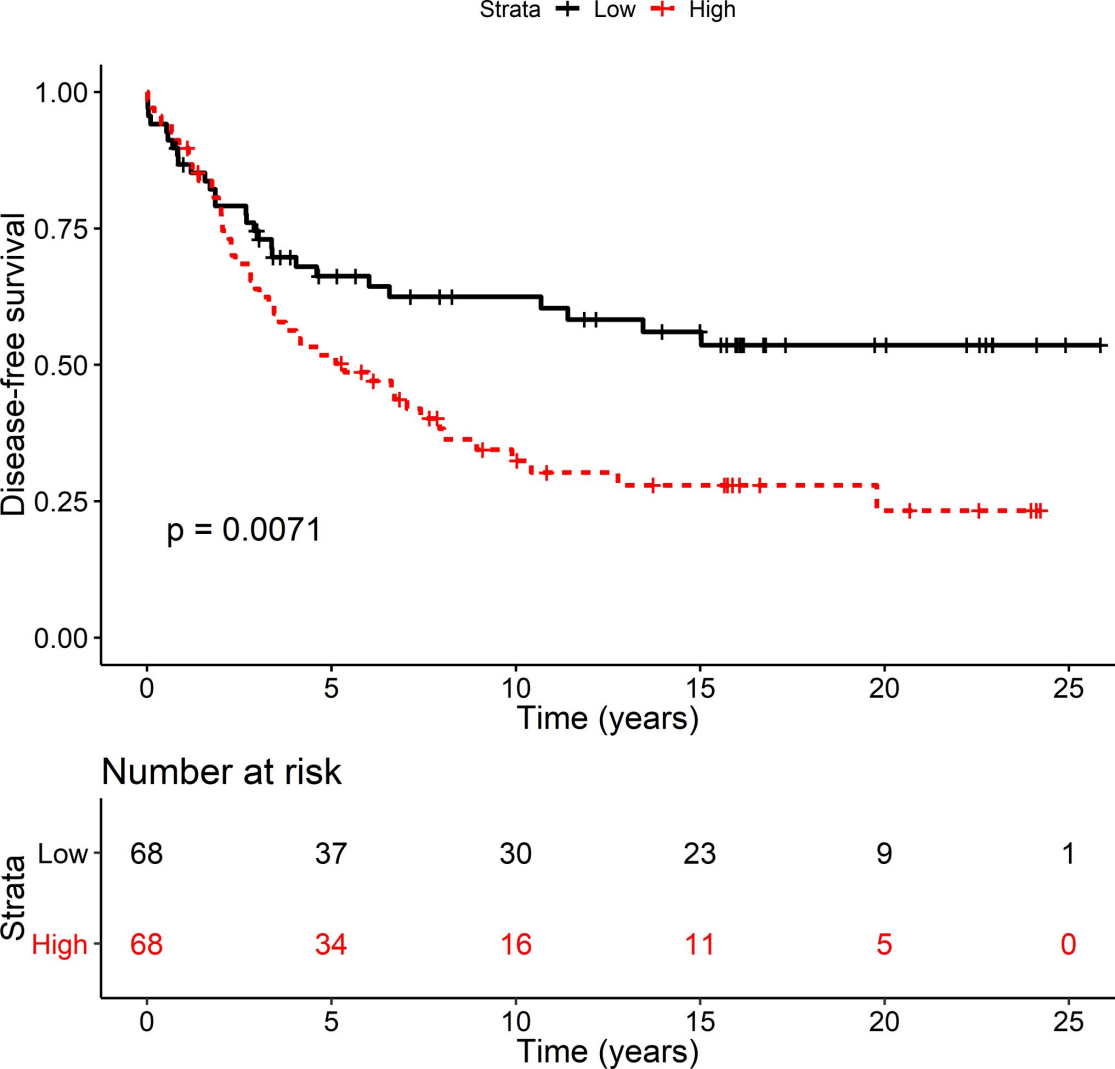
High miR-21-3p levels associated with shorter DFS. Disease-free survival (DFS) was examined in cohort-1, an exploration cohort. Patients were divided into two groups based on median expression of miR-21-3p; high reflects above the median expression (red) and low reflects below median expression (black). The log rank p-value was 7.1^.^10^**−3**^. The number of patients at risk at the indicated time point is shown in a table below the graph. The DFS HR was 1.89 (CI 1.18–3.04) and after adjusting for HER2 the HR was 1.72 (CI 1.08–2.78).

The relationship between miR-21-3p and clinical and pathological characteristics was first explored in cohort-1, a breast cancer cohort that contains 139 patients. In breast tumors from cohort-1, RT-qPCR measured miR-21-3p expression and the resultant values were used to test whether miR-21-3p expression levels associated with any clinical and pathological characteristics. This analysis showed miR-21-3p expression was significantly higher in breast tumors from patients with metastasis than in patients without (p = 2.1^.^10^−2^). No statistically significant correlation with other clinical and pathological parameters was detected (S2 Table) but, in cohort-1, patients expressing high (above median) levels of miR-21-3p had a significantly shorter disease-free survival (DFS; log rank p = 7.1^.^10^−3^; Fig 1). The effect of miR-21-3p association on DFS was assessed with Cox regression analysis. The HR of miR-21-3p was 1.89 (95% CI 1.18–3.04). Among breast tumors with amplified ERBB2 (the gene that expresses the HER2 receptor), 30% were also amplified for the MIR21 genomic region [31]. Examining the effect of HER2 expression on miR-21-3p associated survival showed that HR was 1.72 (95% CI 1.08–2.78) after adjusting for HER2 expression. This shows that HER2 expression attenuates the association of miR-21-3p with DFS. Nevertheless, the effect remained significant suggesting high miR-21-3p expression in breast tumors affects the recurrence rate of breast cancer. There was a trend towards shorter breast cancer specific survival (BCSS) in cohort-1 with high miR-21-3p levels although not significant (log rank p = 0.16, S2 Fig).

### MiR-21-3p associates with tumor characteristics that indicate worse prognosis

To follow up results in cohort-1, association with clinicopathological characteristics and survival was analyzed in cohort-2 (n = 281), TCGA (n = 946) and METABRIC (n = 1174). In METABRIC, miR-21-3p was highly expressed in HER2-positive tumors (p = 2.63^.^10^−9^), large tumors (> 20 mm, p = 2.0^.^10^−2^), tumors of histologic grade 3 (p = 3.68^.^10^−14^), lymph node-positive tumors (p = 1.0^.^10^−3^), HER2 tumors according to PAM50 classification (p < 2^.^10^−16^), and HER2+ tumors according to the 3-Gene classifier subtype (p < 2^.^10^−16^; S3 Table). Furthermore, miR-21-3p was more highly expressed in ductal than lobular tumors (1.69^.^10^−7^)) and in tumors from patients, who had developed metastasis (p = 2.0^.^10^−2^). Compared to METABRIC, both cohort-2 and TCGA represent fewer tumors and fewer available clinical and pathological parameters. Even so, the data confirmed the association detected in METABRIC. MiR-21-3p values were again significantly higher in HER2-positive tumors in cohort-2 (p = 3.0^.^10^−3^) and in histologic grade 3 tumors (p = 2.6^.^10^−2^; S4 Table). Also, in cohort-2, miR-21-3p was higher in large tumors (> 20 mm, p = 7.8^.^10^−2^) and in tumors from patients with metastasis (p = 6.8^.^10^−2^). In TCGA, miR-21-3p was more highly expressed in HER2-positive tumors (p = 5.82^.^10^−4^) and the HER2-enriched molecular subtype (p = < 2^.^10^−16^; S5 Table). Taken together, these data suggest high miR-21-3p levels associated with clinical and pathological characteristics of worse patient prospects.

### High miR-21-3p expression associates with shorter survival

**Fig 2:**
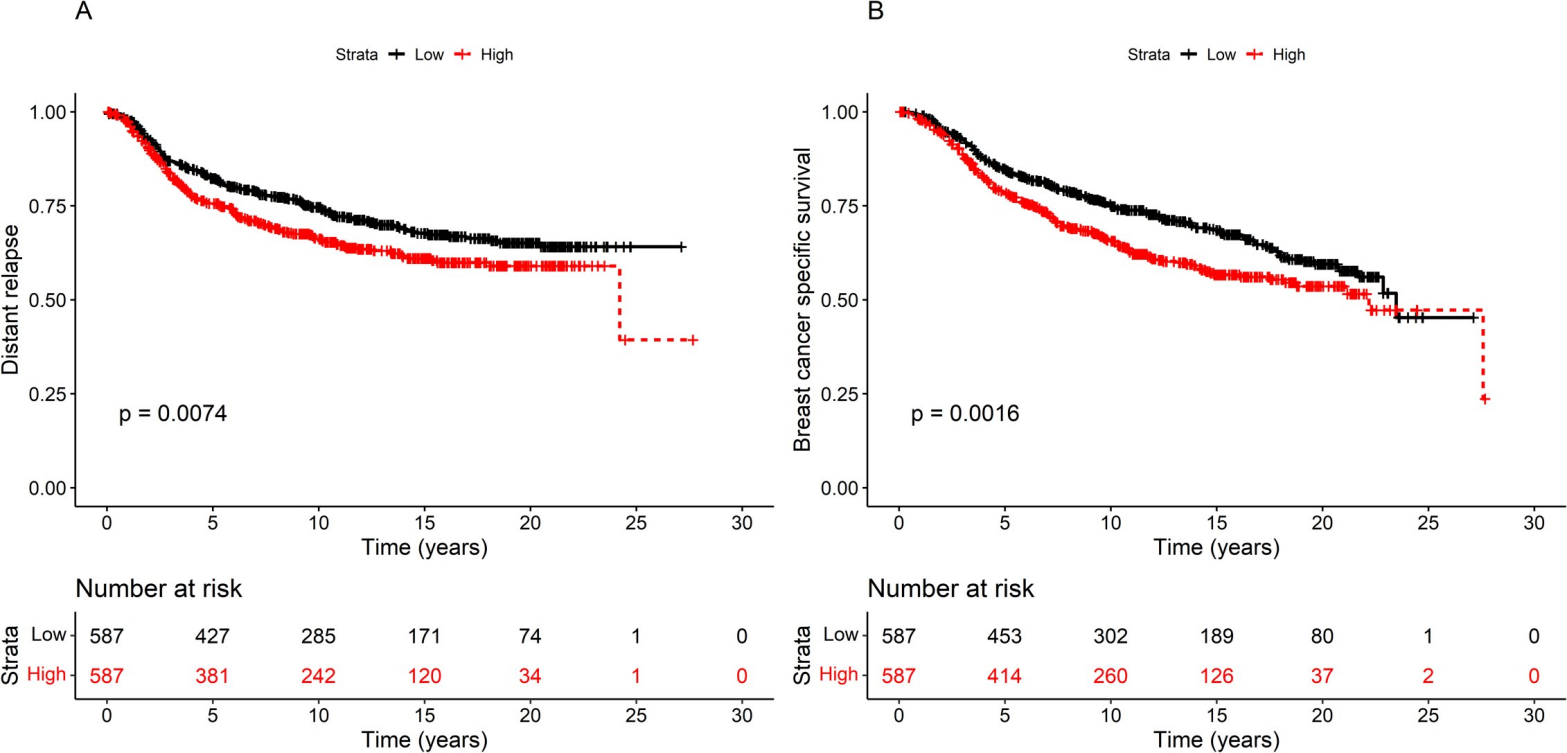
High miR-21-3p expression associated with shorter survival in METABRIC. A) Distant relapse (DR) and B) breast cancer-specific survival (BCSS) was examined in METABRIC, the largest validation cohort. Patients were divided into two groups according to expression of miR-21-3p: above median (red) and below median (black). The DR log rank p-value was 7.4^.^10^−3^ and the BCSS log rank p-value was 1.6^.^10^−3^. The number of patients at risk at each time point is shown in a table below the graph. The BCSS HR was 1.39 (CI 1.15–1.70).

Since malignant tumor characteristics likely predict survival outcomes, BCSS and DFS, as a function of miR-21-3p expression, was calculated for cohort-2, TCGA, and METABRIC. In METABRIC, patients overexpressing miR-21-3p (above median) had significantly shorter distance relapse (DR) and BCSS than those expressing levels below the median (DR: log rank p = 0.016, BCSS: log rank p = 1.6^.^10^−3^, Fig 2). DR in METABRIC is defined as the appearance of a metastasis in distal organs, i.e, not breast, which is not the equivalent to DFS in cohort-1, which includes locoregional recurrence. In cohort-2, DFS and BCSS was borderline significant (DFS: log rank = 0.06, BCSS: log rank p = 0.06; S3 Fig), while in TCGA there was no association (S3 Fig). Differences in the composition, time of diagnosis and treatment of the cohorts may contribute to the inconsistent results. MiR-21-3p expression was high in grade 3 tumors, large tumors, and tumors with lymph node positivity, all of which predict a poorer outcome. A Cox regression analysis tested whether high miR21-3p expression correlated with shorter survival in METABRIC because of characteristics other than miR-21-3p. The HR of miR-21-3p on BCSS was 1.38 (95% CI 1.13–1.68). Lymph node positivity and high tumor stage attenuated the effect of miR-21-3p slightly, but the largest confounders were HER2 and histologic grade (Table 1). If HER2 positivity was taken into account, then the HR of miR-21-3p was reduced to 1.28 (95% CI 1.05–1.57), and with histologic grade the HR of miR-21-3p decreased to 1.23 (95% CI 1.01–1.51). Nevertheless, even after adjusting for confounding variables, the effect of miR-21-3p on survival remained, suggesting high levels of miR-21-3p contribute to a worse prognosis.

**Table 1:** Adjustment of BCSS in METABRIC (n = 1174) for confounding variables.

	HR	CI	p-value
miR-21-3p	1.377	1.127–1.681	2.0^.^10^−3^
+HER2	1.284	1.048–1.574	1.6^.^10^−2^
+ER	1.336	1.093–1.663	5.0^.^10^−3^
+PR	1.336	1.094–1.633	5.0^.^10^−3^
+age^1^	1.377	1.128–1.682	2.0^.^10^−3^
+tumor size	1.416	1.158–1.731	7.0^.^10^−4^
+nodes	1.319	1.080–1.611	7.0^.^10^−3^
+ grade	1.231	1.006–1.508	4.4^.^10^−2^
+VMP1^1^	1.470	1.179–1.833	6.0^.^10^−4^
+RPS6KB1^1^	1.385	1.129–1.700	2.0^.^10^−3^
+PPM1D^1^	1.403	1.146–1.718	1.0^.^10^−3^
+BCAS3	1.375	1.126–1.679	2.0^.^10^−3^
+miR-21-5p^1^	1.377	1.126–1.684	2.0^.^10^−3^

^1^Continuous variables.

### MiR-21-3p is more highly expressed in breast tumors than normal breast tissue

One indication that miR-21-3p plays a role in tumor progression might be a difference in expression level when comparing normal and tumor tissue. To test this, RT-qPCR measured miR-21-3p levels, comparing breast tumor to normal breast tissue, in samples from 35 patients from cohort-2. MiR-21-3p expression was significantly higher in tumors (paired t-test, p = 4.50^.^10^−13^, S4 Fig), a result confirmed by comparing miR-21-3p expression levels in 172 breast tumors from The Cancer Genome Atlas (TCGA) with matched normal breast tissue (p = 1.10^.^10^−15^, S4 Fig).

### Genes co-amplified with miR-21-3p did not attenuate its effect on survival

MIR21 is located at 17q23.1 (GRCh38), a region frequently amplified in breast tumors. Depending on the tumor’s histological origin, this region can be amplified in up to 22% of primary breast tumors [32]. Genes in amplified regions are sometimes overexpressed, which can support tumor development. High expression from genes neighboring MIR21 has been reported in breast tumors [33]. MIR21 neighboring genes within the refined 17q23.1 amplicon are CLTC, PTRH2, VMP1, TUBD1, RPS6KB1 and RNFT1. PPM1D and BCAS3 are included within a region of gain bordering the amplicon. Of these genes, only RPS6KB1, VMP1, PPM1D and BCAS3 have been implicated in tumor development using clinical data from breast cancer patients [18, 34–36]. Therefore, we performed correlation analyses with data from METABRIC to examine whether miR-21-3p was highly expressed in concurrence with VMP1, RPS6KB1, PPM1D and BCAS3. Expression of miR-21-3p significantly correlated with RPS6KB1 (r = 0.38, p < 2.2^.^10^−16^), VMP1 (r = 0.57, p < 2.2^.^10^−16^), PPM1D (r = 0.31, p< 2.2^.^10^−16^), and BCAS3 (r = 0.07, p = 1.66 ^.^10^−2^) (S5 Fig). Even so, high expression from these genes did not attenuate miR-21-3p’s effect on survival (Table 1). The correlation with miR-21-5p was analyzed as well. In the METABRIC cohort, there was a significant correlation between miR-21-3p and miR-21-5p expression (r = 0.1, p = 4.89^.^10^−4^; S5 Fig). Nevertheless, the elevation of miR-21-5p expression level was incremental and the effect of miR-21-3p on survival was not confounded by miR-21-5p (Table 1).

### MiR-21-3p down-regulates potential tumor suppressor genes

**Fig 3:**
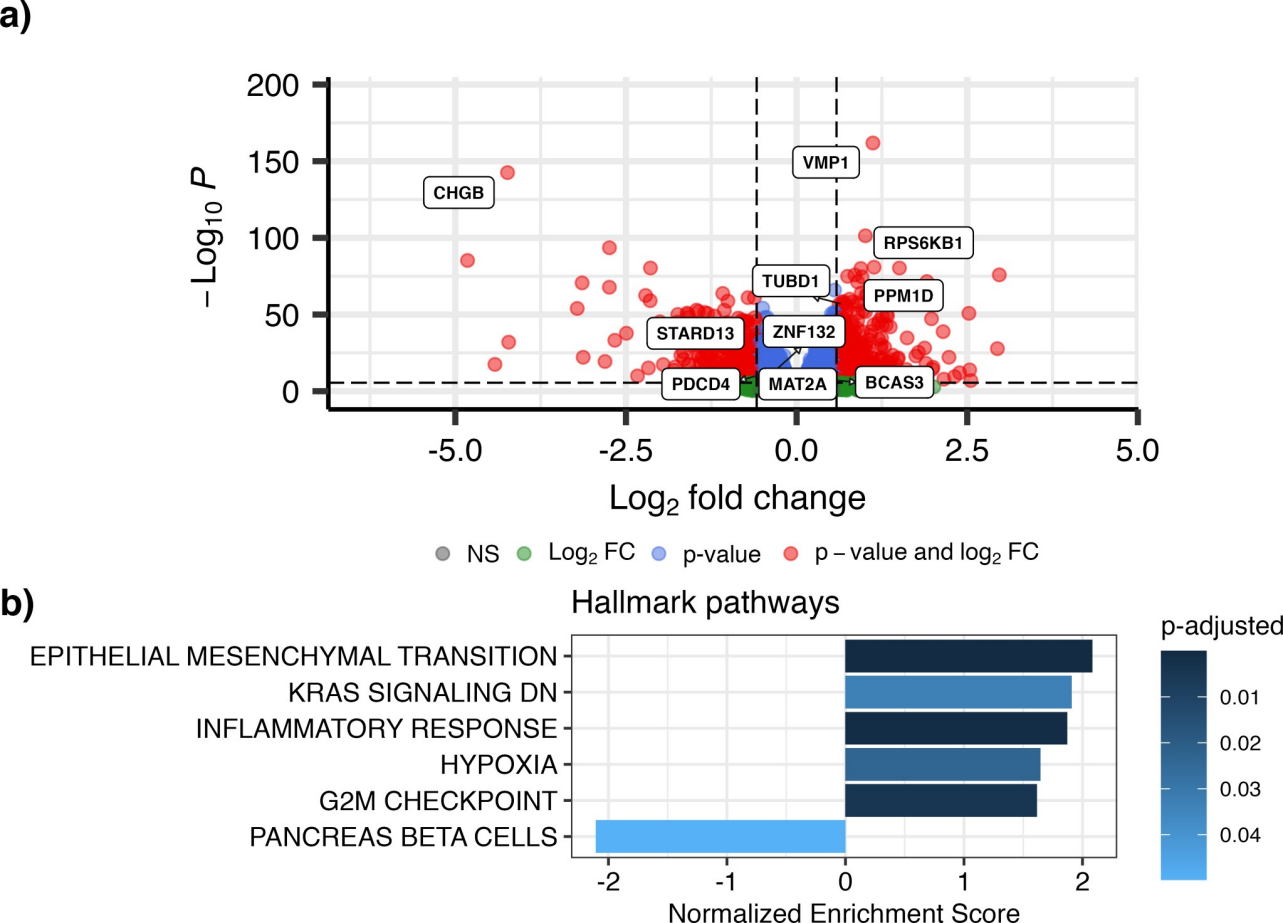
Meta-analysis of mRNA correlation with miR-21-3p. a) Volcano plot from TCGA and METABRIC meta-analysis, showing genes in red that had a significant p-value (FWER threshold) and at least 1.5 fold change in expression (|log_2_(FC)|>0.585). Select genes relevant to this research are highlighted. b) Gene set enrichment analysis (GSEA) of the Hallmark pathways, obtained from the MSigDB collections, showed that genes in the meta-analysis that positively correlate with miR-21-3p expression (using 5% FWER as significance threshold from the meta-analysis while only including genes that were significantly correlated with miR-21-3p in both TCGA and METABRIC at 5% FDR) fall within pathways of EMT, proliferation and inflammation while genes that negatively correlate with miR-21-3p are in the pancreas beta cells pathway.

MiRs bind their target mRNAs, destabilizing them which results in their degradation. To identify targets of miR-21-3p, we conducted a correlation analysis in the METABRIC and TCGA cohorts, and combined the results in a meta-analysis using Stouffer’s method. A volcano plot of the results (~12,400 genes), showing fold change (log_2_(FC)) and the significance values (negative log_10_P) was plotted (Fig 3A). In total, we identified 853 down-regulated genes and 1,822 up-regulated genes at 5% FWER for the meta-analysis and further requiring that each gene also be significantly correlated with miR-21-3p in each cohort (using the less stringent 5% FDR for significance cut-off).

Not surprisingly, the upregulated gene that correlated most significantly with miR-21-3p was VMP1 (up- and down-regulated genes are listed in S6 Table). Gene set enrichment analysis (GSEA) found miR-21-3p expression positively correlated most with its neighboring genes within the amplicon at 17q23.1, namely Farmer´s cluster 5 (p-adjusted = 2^.^10^−23^; [37]) and genes in amplicon 17q21-25 (p-adjusted = 1^.^10^−21^; [38]; S7 Table). GSEA of the Hallmark pathways identified pathways that support proliferation, the epithelial-to-mesenchymal transition (EMT), and responses to inflammation (Fig 3B), all of which play pivotal roles in cancer progression (S8 Table). Since miR-21-3p expression correlated with expression of its neighboring genes, these data cannot distinguish whether the downstream effects are due to miR-21-3p or neighboring genes. However, miR-21 has been shown to affect growth and EMT in breast cancer cell lines [39–41].

Gene ontology (GO) gene sets that correlated significantly with genes that inversely correlate with miR-21-3p include metabolic processes, transmembrane transport, and cilium organization. Cilium organization is a key signaling hub, for example in Wnt and MAPK signaling, and plays a role in cancer [42] (S9 Table). The most down-regulated gene from the meta-analysis was chromogranin-B (CHGB), which is associated with malignancy and metastasis in pancreatic neuroendocrine tumors (PNETs) [43]. Lower expression of CHGB was reported in invasive ductal carcinoma of the breast as compared to non-invasive ductal carcinoma [44]; and breast cancer patients with CHGB negative tumors have poorer prognosis than those with CHGB positive tumors [45].

**Fig 4:**
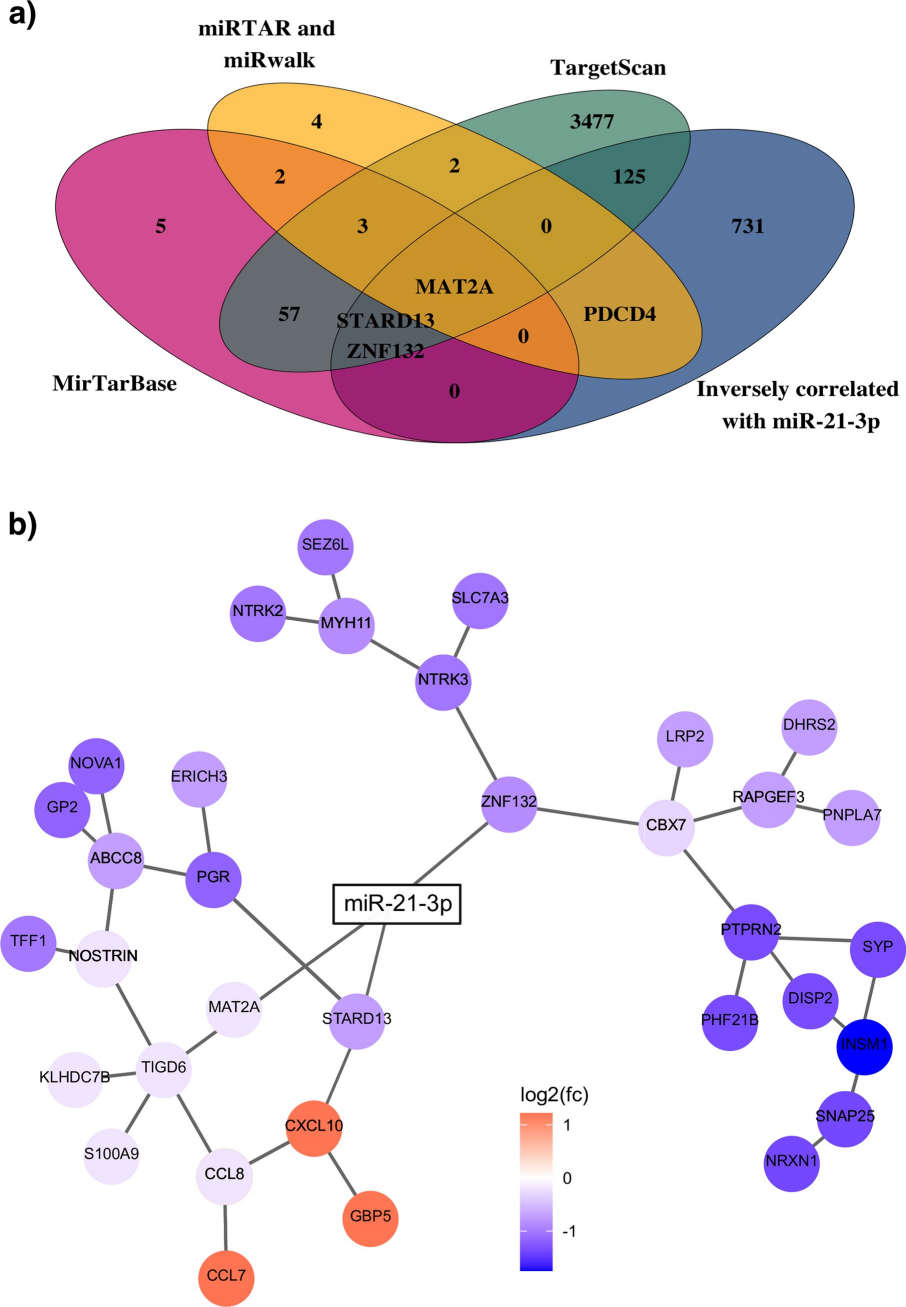
MiR-21-3p validated targets viewed in a network. a) Venn diagram showing overlap between the genes identified in the analysis as inversely correlated with miR-21-3p (blue) and miRNA target databases miRTarBase (red), TargetScan (green), miRTAR and miRwalk (yellow). 129 genes from our analysis are listed as predicted targets of miR-21-3p in these databases out of which three, STARD13, ZNF132 and MAT2A, have been validated experimentally. MAT2A was the only gene identified in all four databases. b) Simplified gene co-expression network diagram showing the three validated targets of miR-21-3p. The network diagram was constructed using genes with expression that significantly correlated with miR-21-3p levels in the meta-analysis as the nodes. The color represents the mean of the log_2_(FC) from TCGA and METABRIC. The edges between gene nodes were predicted by applying the ARACNe-AP algorithm to breast cancer samples from TCGA.

To identify direct mRNA targets of miR-21-3p, predicted targets from the MirTarBase, miRWalk and TargetScan were compared to the differentially expressed genes from the meta-analysis (Fig 4A). Our analysis revealed 129 potential targets of miR-21-3p that overlapped with genes in METABRIC and TCGA, and were inversely correlated with miR-21-3p. Among these was PDCD4, a previously described miR-21-5p target.

Since each of the three databases suggested different direct targets of miR-21-3p, we focused on targets that were experimentally validated and limited our analysis to miR-21-3p targets from miRTarBase. According to this analysis, eight of the 70 genes were shared; and among these miR-21-3p inversely correlated with three: STARD13, MAT2A and ZNF132. STARD13 is a tumor suppressor that plays a role in breast cancer invasion and metastasis [46, 47]. ZNF132 is implicated as a master transcriptional regulator of networks that underlie the breast cancer phenotype [48]. Additionally, high MAT2A expression predicts shorter distant metastasis-free survival in ER positive patients [49]. A network analysis identified downstream genes that might be regulated by miR-21-3p. The network edges are based on co-expression of genes from the BRCA-TCGA data analyzed using the ARACNe-AP algorithm [50]; and the nodes are selected from the lowest p-values in the meta-analysis. Collectively, these genes fall within a network that is important for cell proliferation, regulation of apoptosis, and cell migration (Fig 4B). Taken together, these results indicate miR-21-3p expression supports activation of pathways that facilitate tumor progression when their control is deregulated. Although our results are supported by some experimental evidence, further validation is needed.

## Discussion

In our study, we demonstrated that high levels of miR-21-3p associated with pathoclinical characteristics of a worse prognosis and shorter BCSS. In addition, we verified that the miR-21-3p target genes, ZNF132, STARD13, and MAT2A, were significantly down-regulated when miR-21-3p expression was high. These genes are implicated in breast tumor development.

MiR-21 is up-regulated in many cancer types and can be up-regulated in several ways. It has its own promoter in intron 10 of VMP1, at 17q23.1 (see Fig 1 in a review by Bautista-Sánchez et al. [6]). Its locus is frequently amplified in breast tumors, which can increase expression of the genes therein. If VMP1 is the 3´ partner of a fusion gene, then mature miR-21 products increase [51]. VMP1-miR-21 fusion transcripts are known [2, 4, 49] that result in increased miR-21 products. The locus appears to be regulated by a complex regulatory mechanism, akin to the situation in colorectal cancer where an autoregulatory loop is between miR-21 and VMP1 [52] and a miR-21-3p isomiR is suggested to downregulate miR-21-5p [53]. Our data do not support downregulation of miR-21-5p by miR-21-3p (S5 Fig) but they do not preclude a different scenario in particular breast cancer subtypes. MiR-21-3p reportedly is more abundant in tumors, including breast tumors [14, 15], than in normal tissue, an effect we confirmed in the cohorts we analyzed.

Our results suggest elevated expression of miR-21-3p might serve as a prognostic marker in breast cancer. In the exploration cohort (cohort-1), miR-21-3p levels associated with shorter survival. Among the validation cohorts, high miR-21-3p levels also affected BCSS, with significance in the METABRIC cohort, a trend detected in cohort-2 but not in TCGA. This discrepancy between cohorts may be due to the treatments received by patients comprising each cohort, despite that their clinical and pathological characteristics were similar they differed in their time at diagnosis, which in turn affects treatment. Apart from receiving drug combinations that targeted either pathways controlled by the hormone receptors or proliferation (chemotherapy), the main difference between the cohorts was treatment with trastuzumab (Herceptin). Trastuzumab binds to and inhibits the activity of the HER2 receptors and as a result reduces the activity of signaling pathways that control cellular growth and survival [54, 55]. TCGA breast cancer patients and cohort-2 received trastuzumab whereas patients in METABRIC and cohort-1 did not. This difference may account for some of the discrepancy since HER2 was one of the main confounding factors. Moreover, technical reasons might account for some differences, e.g. the techniques that measure miR-21-3p (see methods). Further complicating the analysis, a variety of miRs isoforms (called isomiRs) were identified in colorectal cancers [53]; and the probes we used to analyze cohorts 1 and 2 only captured the reference isomiR. In contrast, the METABRIC microarray probe is 15 nucleotides and the probe sequence is embedded in the reference sequence. Since detection is based on hybridization, the METABRIC analysis has the potential to capture additional isomiRs. In TCGA, the miR-21-3p values used in this study represent nine isomiRs including the reference (S1 Fig). These considerations are important as isomiRs affect the cellular transcriptome differently and can be differently expressed based on ethnicity [56].

The function of miR-21-3p may depend on tumor type and context. In non-small cell lung cancer (NSCLC) and esophageal squamous cell carcinoma (ESCC), miR-21-3p was more highly expressed in tumors than the adjacent normal tissue [57, 58]. In ESCC, high miR-21-3p levels also associated with a high risk of cancer progression [58], in agreement with our findings. In cell-based assays in a colorectal cancer cell line [59] and in ovarian and prostate cell lines [60], high miR-21-3p had oncogenic properties [59]; however, in a hepatocellular cancer cell line, miR-21-3p overexpression suppressed growth and increased apoptosis, suggesting tumor suppressive properties [61]. One breast cancer study focusing on miR-21-3p is in agreement with the results in the hepatocellular cancer cell line, as low levels of miR-21-3p were a risk factor for overall survival (OS) in patients with triple-negative breast tumors [14]. Another breast cancer study suggested that high miR-21-3p was a potential biomarker for early detection of breast cancer [16]. We did not analyze miR-21-3p expression levels with respect to early breast cancer in our study but our data are in line with these results as they suggest that high miR-21-3p is a prognostic factor for BCSS. Depending on the cellular context, the particular miR-21-3p isomiR expressed [53], and the target gene(s), miR-21-3p may act as either a tumor suppressor or oncogene.

To gain insight into the biological role of miR-21-3p, we looked for genes with expression that increased with high miR-21-3p, and whether these genes associated with cellular pathways active in tumor progression. Unsurprisingly, some of the genes that most significantly correlated with high miR-21-3p were its genetic neighbors at 17q23.1. These genes are not confounders in the survival analysis but their effect is difficult to separate from that of miR-21-3p. The significantly upregulated Hallmark pathways, such as the epithelial-to-mesenchymal transition, G2/M checkpoint control, and inflammatory response (Fig 3B) are well known to be active in cancer [62].

To identify direct targets of miR-21-3p, miRTarBase was used because it includes experimentally validated targets. The three targets—STARD13, ZNF132 and MAT2A—that were significantly down-regulated when miR-21-3p expression was high are genes already implicated in breast cancer [46–49]. STARD13 reportedly functions in cytoskeletal reorganization, proliferation, and motility, all of which are processes necessary for cancer progression [46]. Silencing of the transcription factor ZNF132 promotes progression in ESCC [63]; and its downregulation is associated with poor prognosis in prostate cancer [64], indicating tumor suppressive properties. MAT2A, Methionine Adenosyltransferase 2A, catalyzes the production of S-adenosylmethionine, which is important for most cellular processes. In contrast to ZNF132 and STARD13, upregulation of MAT2A is associated with poor prognosis [49].

A genetic network that expands from the three miR-21-3p targets we validated and are significantly downregulated includes genes implicated in processes known to affect cancer progression (Fig 4B). Among the genes in the ZNF132 node, CBX7 is implicated in cancer progression and EMT [65], PTPRN2 confers resistance to apoptosis [66], and NTRK3 is a receptor tyrosine kinase whose overactive kinase domain is implicated in growth and metastasis [67]. The STARD13 node includes the progesterone receptor PGR, which is interesting because high levels of its isoforms induce invasion and metastasis [68] and high PGR also indicates a good prognosis in ER-positive breast cancer [69]. ABCC8, a member of the MRP family involved in multidrug resistance, has a role in diabetes and recently expression patterns of the ABC family were suggested to be new hallmarks of cancer [70]. CXCL10 is a chemokine that, among others, aids immune cells in infiltration of tumors [71]. Overexpression of NOSTRIN, in pancreatic cancer, suppresses migration and invasion [72]. The MAT2A node includes TIGD6, which has not been associated with cancer. Notably, most genes in the network have been linked to cancer progression.

Previous studies identified miR-21-3p target genes: in ovarian cells it targets NAV3 [73], a known tumor suppressor; in hepatocellular carcinoma, it targets SMAD7, an inhibitor of the TGFβ pathway [74]; and in ESCC, it targets TRAF4 [58]. Conversely, miR-21-3p expression upregulates L1CAM, which promotes cell motility, invasion, metastasis, and chemoresistance [75]. L1CAM and NAV3 are not targets of miR-21-5p [73, 75], but among other well-known targets of miR-21-5p [76], our study identified PDCD4 as being regulated by miR-21-3p as well. Although miR-21-3p might affect the same pathways as miR-21-5p, (e.g., invasion and metastasis), our data suggest it does so, at least in part, through targeting different genes. Indeed our data shows miR-21-3p is a prognostic marker in breast cancer independent of oncomiR miR-21-5p. These results highlight the importance of studying each strand of a mature miRNA (i.e., the 3p and the 5p), independently, to distinguish each component’s biological function. In the case of miR-21 much effort has been put into studying miR-21-5p, yet our results indicate miR-21-3p also modulates breast cancer progression.

## Conclusions

Using mRNA, miRNA, clinical, pathological and survival data from a selection of breast cancer patient cohorts, we identified miR-21-3p as a candidate prognostic marker for breast cancer that is associated with shorter breast cancer survival. It is inversely correlated with STARD13, ZNF132 and MAT2A, which are implicated in tumor development. Therefore, this interesting breast cancer candidate miR-21-3p warrants further investigation to fully understand its impact on breast cancer progression.

## Supporting information

S1 Fig Nine miR-21-3p isomiRs in the BRCA cohort from TCGA are significantly higher in tumor than in matched normal tissue. (PDF)

S2 Fig High miR-21-3p levels do not associate with BCSS. (PDF)

S3 Fig Disease-free and breast cancer specific survival in cohort-2 and TCGA. (PDF)

S4 Fig MiR-21-3p levels were higher in breast tumors than paired normal breast tissues. (PDF)

S5 Fig MiR-21-3p expression levels correlated with PPM1D, VMP1 and RPS6KB1. (PDF)

S1 Table IsomiRs used from TCGA based on significantly higher expression in tumor compared to normal tissue. (PDF)

S2 Table Clinical and pathological characteristics of cohort-1. (PDF)

S3 Table Clinical and pathological characteristics of METABRIC BC cohort. (PDF)

S4 Table Clinical and pathological characteristics of cohort-2. (PDF)

S5 Table Clinical and pathological characteristics of TCGA BC cohort. (PDF)

S6 Table Meta-analysis from TCGA and METABRIC gene expression data. (XLSX)

S7 Table GSEA from curated gene sets (c2) for genes that positively correlate with miR-21-3p. (XLSX)

S8 Table Pathway analysis on the hallmark gene sets using R package fgsea for genes correlated with miR-21-3p. (XLSX)

S9 Table GO analysis using R package enrichGO for genes inversely correlated with miR-21-3p. (XLSX)
