# Supplementary material for: Hsa-miR-21-3p associates with breast cancer patient survival and targets genes in tumor suppressive pathways
Source: PLoS One. 2021 Nov 19;16(11):e0260327. doi: 10.1371/journal.pone.0260327 (PMC8604322; doi:10.1371/journal.pone.0260327)
Supplement: S1 Fig — (PDF) [file pone.0260327.s001.pdf]

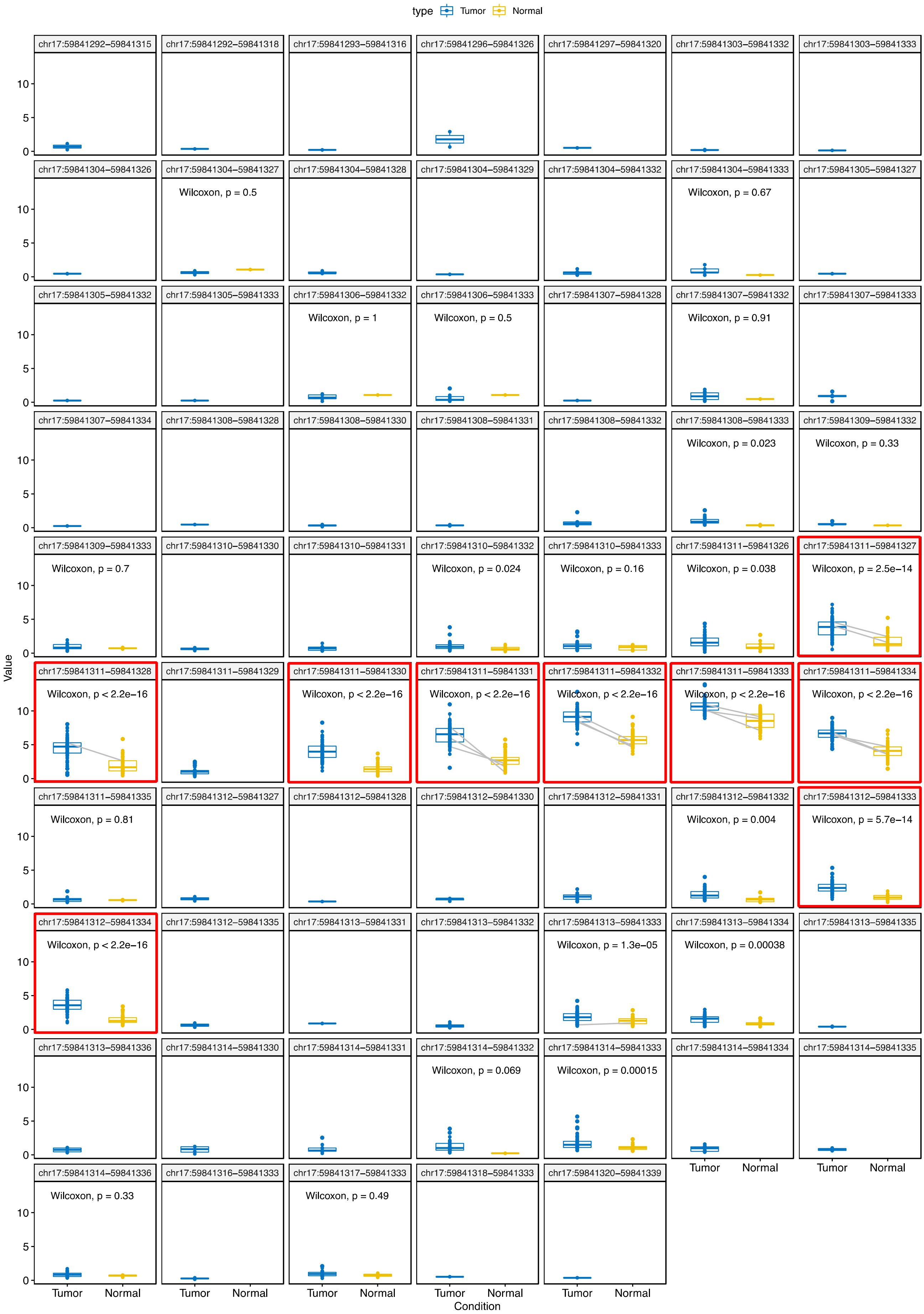
type Tumor Normal
Value
Condition
Tumor
Normal
chr17:59841292–59841315
chr17:59841292–59841318
chr17:59841293–59841316
chr17:59841296–59841326
chr17:59841297–59841320
chr17:59841303–59841332
chr17:59841303–59841333
chr17:59841304–59841326
chr17:59841304–59841327
Wilcoxon, p = 0.5
chr17:59841304–59841328
chr17:59841304–59841329
chr17:59841304–59841332
chr17:59841304–59841333
Wilcoxon, p = 0.67
chr17:59841305–59841327
chr17:59841305–59841332
chr17:59841305–59841333
chr17:59841306–59841332
Wilcoxon, p = 1
chr17:59841306–59841333
Wilcoxon, p = 0.5
chr17:59841307–59841328
chr17:59841307–59841332
Wilcoxon, p = 0.91
chr17:59841307–59841333
chr17:59841307–59841334
chr17:59841308–59841328
chr17:59841308–59841330
chr17:59841308–59841331
chr17:59841308–59841332
chr17:59841308–59841333
Wilcoxon, p = 0.023
chr17:59841309–59841332
Wilcoxon, p = 0.33
chr17:59841309–59841333
Wilcoxon, p = 0.7
chr17:59841310–59841330
chr17:59841310–59841331
chr17:59841310–59841332
Wilcoxon, p = 0.024
chr17:59841310–59841333
Wilcoxon, p = 0.16
chr17:59841311–59841326
Wilcoxon, p = 0.038
chr17:59841311–59841327
Wilcoxon, p = 2.5e–14
chr17:59841311–59841328
Wilcoxon, p < 2.2e–16
chr17:59841311–59841329
chr17:59841311–59841330
Wilcoxon, p < 2.2e–16
chr17:59841311–59841331
Wilcoxon, p < 2.2e–16
chr17:59841311–59841332
Wilcoxon, p < 2.2e–16
chr17:59841311–59841333
Wilcoxon, p < 2.2e–16
chr17:59841311–59841334
Wilcoxon, p < 2.2e–16
chr17:59841311–59841335
Wilcoxon, p = 0.81
chr17:59841312–59841327
chr17:59841312–59841328
chr17:59841312–59841330
chr17:59841312–59841331
chr17:59841312–59841332
Wilcoxon, p = 0.004
chr17:59841312–59841333
Wilcoxon, p = 5.7e–14
chr17:59841312–59841334
Wilcoxon, p < 2.2e–16
chr17:59841312–59841335
chr17:59841313–59841331
chr17:59841313–59841332
chr17:59841313–59841333
Wilcoxon, p = 1.3e–05
chr17:59841313–59841334
Wilcoxon, p = 0.00038
chr17:59841313–59841335
chr17:59841313–59841336
chr17:59841314–59841330
chr17:59841314–59841331
chr17:59841314–59841332
Wilcoxon, p = 0.069
chr17:59841314–59841333
Wilcoxon, p = 0.00015
chr17:59841314–59841334
chr17:59841314–59841335
chr17:59841314–59841336
Wilcoxon, p = 0.33
chr17:59841316–59841333
chr17:59841317–59841333
Wilcoxon, p = 0.49
chr17:59841318–59841333
chr17:59841320–59841339

**S1 Fig. Nine miR-21-3p isomiRs in the BRCA cohort from TCGA are significantly higher in tumor than in matched normal tissue.**

Expression of all distinct miR-21-3p isomiRs in BRCA-TCGA data from matched tumor and normal samples. The isomiRs marked with a red box are the ones included in the analysis. Their chromosomal location according to hg38 is given in S1 Table. The tumor samples are shown in blue and the normal samples in yellow.
