# Supplementary material for: Hsa-miR-21-3p associates with breast cancer patient survival and targets genes in tumor suppressive pathways
Source: PLoS One. 2021 Nov 19;16(11):e0260327. doi: 10.1371/journal.pone.0260327 (PMC8604322; doi:10.1371/journal.pone.0260327)
Supplement: S2 Fig — (PDF) [file pone.0260327.s002.pdf]

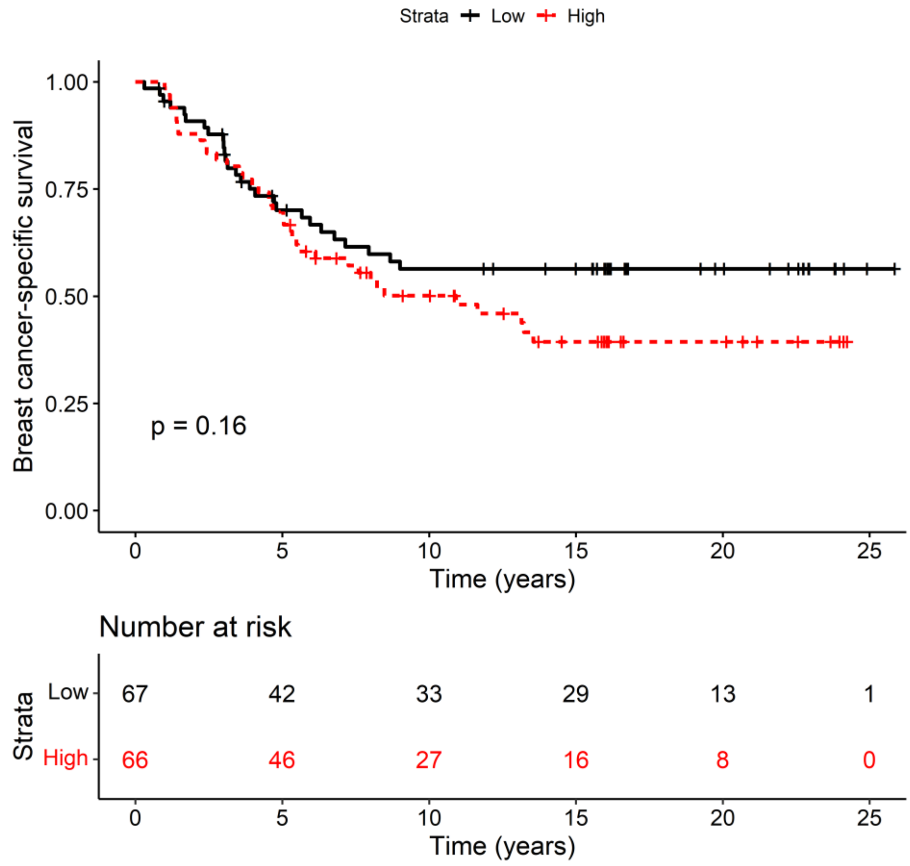

S2 Fig. High miR-21-3p levels do not associate with BCSS.

Breast cancer specific survival (BCSS) was examined in cohort-1. Patients were divided into two groups based on median expression of miR-21-3p; high reflects above the median expression (red) and low reflects below median expression (black). The log rank p-value was 0.16. The number of patients at risk at the indicated time point is shown in a table below the graph.
