# Supplementary material for: Hsa-miR-21-3p associates with breast cancer patient survival and targets genes in tumor suppressive pathways
Source: PLoS One. 2021 Nov 19;16(11):e0260327. doi: 10.1371/journal.pone.0260327 (PMC8604322; doi:10.1371/journal.pone.0260327)
Supplement: S3 Fig — (PDF) [file pone.0260327.s003.pdf]

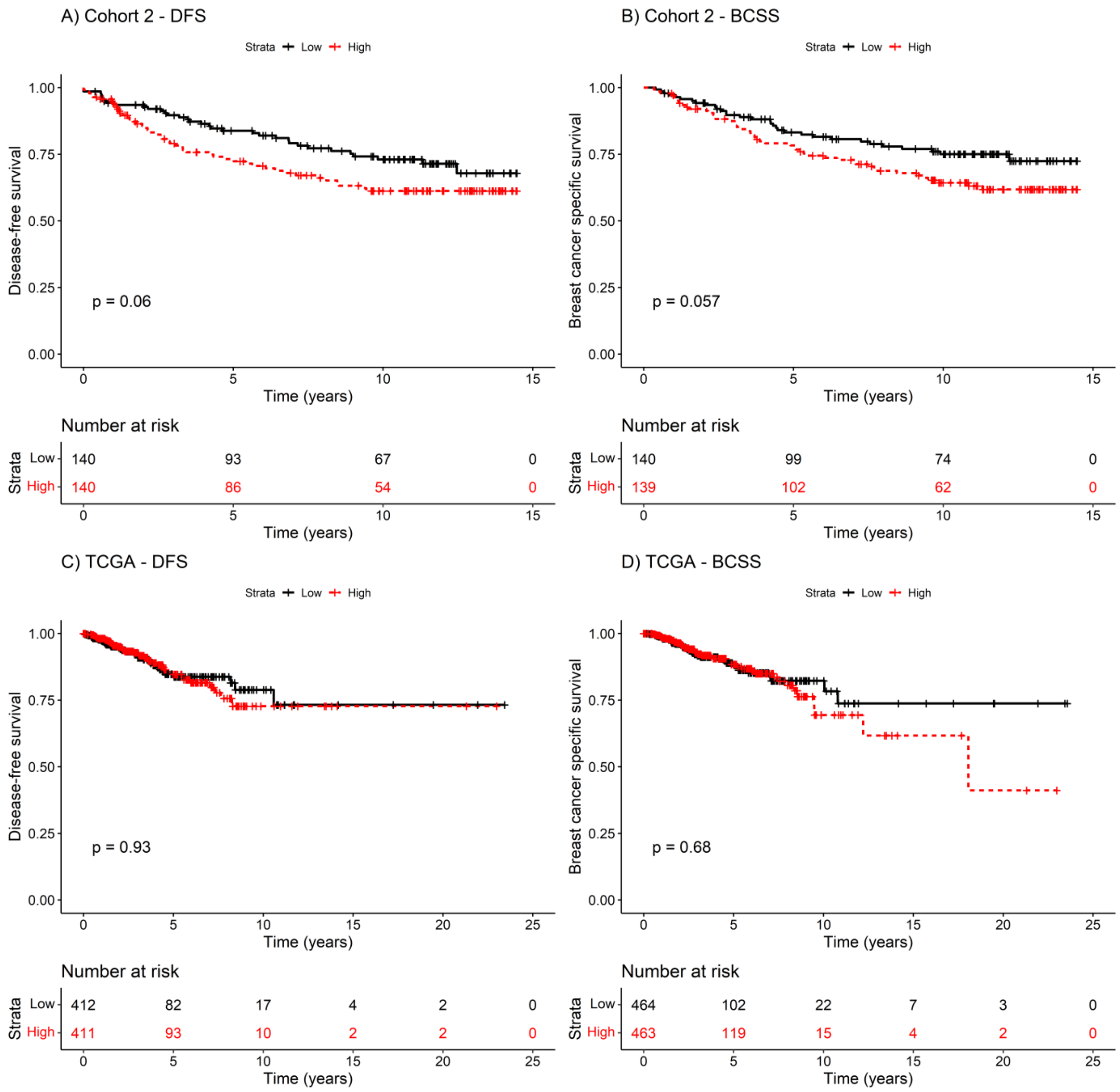

S3 Fig. Disease-free and breast cancer specific survival in cohort-2 and TCGA.

A and C) Disease-free survival (DFS) and (B and D) breast cancer specific survival (BCSS) was examined in (A and B) cohort-2 and (C and D) TCGA. MiR-21-3p expression was used to divide the patients into two groups, high (red) and low (black) based on the median expression levels of miR-21-3p. The log rank p-values are shown in the graphs. The numbers of patients at risk at each time point are shown in tables below the graphs.
