# Supplementary material for: Hsa-miR-21-3p associates with breast cancer patient survival and targets genes in tumor suppressive pathways
Source: PLoS One. 2021 Nov 19;16(11):e0260327. doi: 10.1371/journal.pone.0260327 (PMC8604322; doi:10.1371/journal.pone.0260327)
Supplement: S4 Fig — (PDF) [file pone.0260327.s004.pdf]

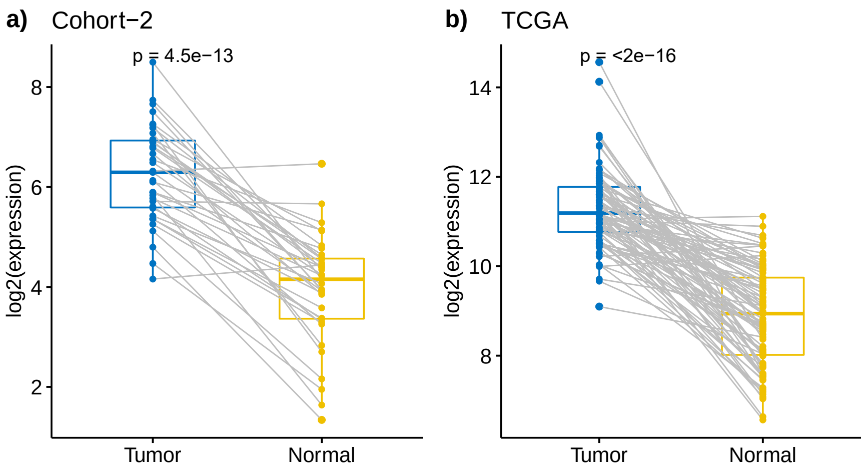

S4 Fig. MiR-21-3p levels were higher in breast tumors than paired normal breast tissues.

MiR-21-3p was examined in breast tumors and normal breast tissue from a) cohort-2 (n = 35) and b) TCGA (n = 172). A paired t-test was used to analyze expression levels between tumors and normal tissue. Expression in tumors was significantly higher than in normal breasts in cohort-2, $p = 4.5 \cdot 10^{-13}$ and TCGA, $p < 2 \cdot 10^{-16}$.
