# Supplementary material for: Hsa-miR-21-3p associates with breast cancer patient survival and targets genes in tumor suppressive pathways
Source: PLoS One. 2021 Nov 19;16(11):e0260327. doi: 10.1371/journal.pone.0260327 (PMC8604322; doi:10.1371/journal.pone.0260327)
Supplement: S5 Fig — (PDF) [file pone.0260327.s005.pdf]

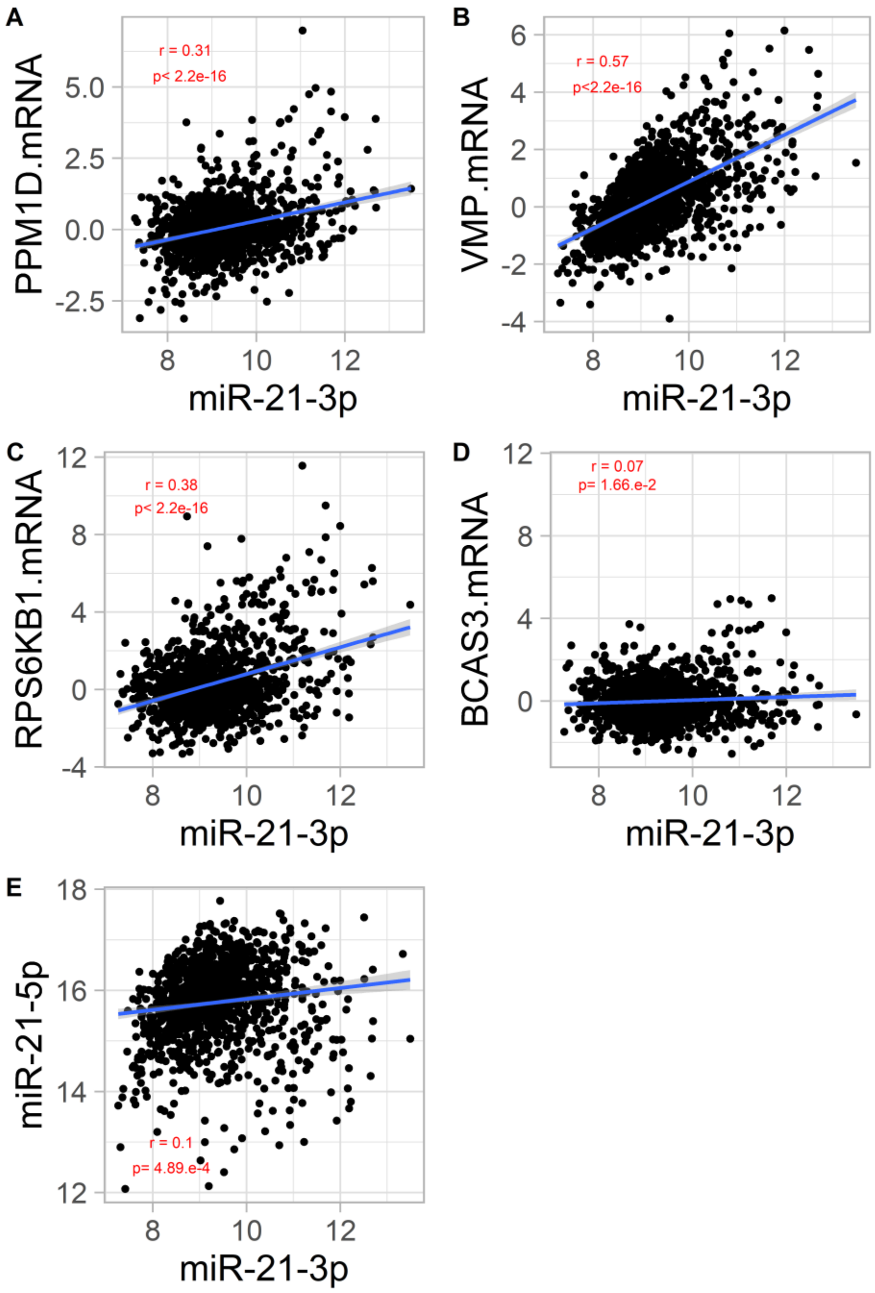

S5 Fig. MiR-21-3p expression levels correlated with PPM1D, VMP1 and RPS6KB1.

MiR-21-3p levels in METABRIC (measured via Agilent microarray) correlated with PPM1D mRNA, VMP1 mRNA, RPS6KB1 mRNA and BCAS3 mRNA (measured by the same technique). (A) PPM1D and miR-21-3p Pearson r value was 0.31, $p < 2.2 \cdot 10^{-16}$; (B) VMP1 and miR-21-3p Pearson r value was 0.57, $p < 2.2 \cdot 10^{-16}$; (C) RPS6KB1 and miR-21-3p Pearson r value was 0.38, $p < 2.2 \cdot 10^{-16}$; (D) BCAS3 and miR-21-3p Pearson r value was 0.07, $p = 1.66 \cdot 10^{-2}$; (E) miR-21-5p and miR-21-3p Pearson r value was 0.11, $p = 4.89 \cdot 10^{-4}$.
