# Supplementary material for: Hsa-miR-21-3p associates with breast cancer patient survival and targets genes in tumor suppressive pathways
Source: PLoS One. 2021 Nov 19;16(11):e0260327. doi: 10.1371/journal.pone.0260327 (PMC8604322; doi:10.1371/journal.pone.0260327)
Supplement: S1 Table — (PDF) [file pone.0260327.s006.pdf]

miR-21-3p isomiRs used from BRCA-TCGA based on significantly higher expression in tumor compared to normal tissue

hg38:chr17:59841311-59841327:+
hg38:chr17:59841311-59841328:+
hg38:chr17:59841311-59841330:+
hg38:chr17:59841311-59841331:+
hg38:chr17:59841311-59841332:+
hg38:chr17:59841311-59841333:+
hg38:chr17:59841311-59841334:+
hg38:chr17:59841312-59841333:+
hg38:chr17:59841312-59841334:+
