# Supplementary material for: Hsa-miR-21-3p associates with breast cancer patient survival and targets genes in tumor suppressive pathways
Source: PLoS One. 2021 Nov 19;16(11):e0260327. doi: 10.1371/journal.pone.0260327 (PMC8604322; doi:10.1371/journal.pone.0260327)
Supplement: S2 Table — (PDF) [file pone.0260327.s007.pdf]

Clinical and pathological characteristics of BRCA cohort-1

	n =139	miR21-3p mRNA<br>median (25 and 75%)	p-value
**Age**			0.263
< 50	53	0.114 (-0.420, 1.277)	
≥ 50	86	-0.157 (-1.007, 0.815)	
**Estrogen receptor**			0.483
Negative	46	-0.261 (-0.988, 0.818)	
Positive	89	0.116 (-0.677, 1.104)	
Unknown	4		
**Progesterone receptor**			0.361
Negative	63	-0.247 (-0.910, 0.801)	
Positive	69	0.1271 (-0.677, 1.201)	
Unknown	7		
**HER2 status**			0.286
Negative	116	-0.041 (-0.921, 1.025)	
Positive	22	0.239 (-0.330, 1.172)	
Unknown	1		
**Tumor size**			0.340
≤ 20	45	0.269 (-0.517, 1.018)	
> 20	94	-0.157 (-0.974, 0.997)	
Unknown	0		
**Histologic Grade**			0.473
1	12	0.130 (-0.439, 1.327)	
2	79	0.042 (-1.021, 1.032)	
3	47	-0.063 (-0.625, 0.774)	
Unknown	1		
**Ki67**			0.247
1	94	0.060 (-0.724, 1.071)	
2	28	-0.354 (-1.166, 0.395)	
3	15	-0.247 (-0.452, 0.750)	
Unknown	2		
**Nodes**			0.573
Negative	55	-0.063 (-0.862, 0.936)	
Positive	67	0.021 (-0.733, 1.186)	
Unknown	17		
**Histology subtype**			0.305
Ductal	120	-0.036 (-0.890, 0.905)	
Ductal_lobular	1	0.854	
Ductal_mucino	1	0.713	
Lobular	11	0.429 (-0.069, 1.927)	
Medullary	4	-0.749 (-1.110, -0.061)	
Metaplastic	0	NA	
Metastasis_ade	1	(-0.997)	
Mucinous	1	(-1.136)	
**Subtype Hu et al.**			0.462
Basal	24	-0.361 (-0.975, 0.430)	
ERBB2	13	0.114 (-0.275, 0.817)	
LumA	42	0.159 (-0.902, 0.969)	

LumB	29	-0.059 (-1.161, 1.104)	
Normal	11	0.384 (-0.443, 2.594)	
unclassified	12	-0.053 (-0.694, 1.324)	
Unknown	6		
**Metastasis**			0.021
M0	62	-0.416 (-1.041, 0.691)	
M1	76	0.307 (-0.358, 1.306)	
Unknown	1		
