# Supplementary material for: Hsa-miR-21-3p associates with breast cancer patient survival and targets genes in tumor suppressive pathways
Source: PLoS One. 2021 Nov 19;16(11):e0260327. doi: 10.1371/journal.pone.0260327 (PMC8604322; doi:10.1371/journal.pone.0260327)
Supplement: S3 Table — (PDF) [file pone.0260327.s008.pdf]

Clinical and pathological characteristics of METABRIC BRCA cohort

			miR21-3p mRNA	p-value
		n = 1174	median (25 and 75%)	
**Age**				0.857
	< 50	271	-0.004 (-0.474, 0.681)	
	≥ 50	903	0.137 (-0.442, 0.550)	
**Estrogen receptor**				0.004
	Negative	266	0.191 (-0.314, 0.719)	
	Positive	908	-0.033 (-0.471, 0.528)	
**Progesterone receptor**				0.124
	Negative	556	0.066 (-0.380, 0.644)	
	Positive	618	-0.054 (-0.493, 0.524)	
**HER2 status**				2.63E-09
	Negative	1026	-0.040 (-0.477, 0.496)	
	Positive	148	0.459 (-0.088, 1.329)	
**Tumor stage**				0.007
	0	1		
	1	347	-0.082 (-0.509, 0.392)	
	2	569	0.058 (-0.383, 0.653)	
	3	96	0.023 (-0.321, 0.664)	
	4	9	0.915 (0.187, 1.021)	
	Unknown	152		
**Tumor size**				0.022
	≤ 20	503	-0.018 (-0.456, 0.489)	
	> 20	658	0.018 (-0.419, 0.675)	
	Unknown	13		
**Histologic Grade**				3.68E-14
	1	104	-0.252 (-0.516, 0.126)	
	2	469	-0.124 (-0.595, 0.314)	
	3	601	0.212 (-0.322, 0.839)	
**Cellularity**				0.016
	Low	130	-0.018 (-0.599, 0.520)	
	Moderate	430	-0.037 (-0.452, 0.525)	
	High	569	0.056 (-0.406, 0.697)	
	Unknown	45		
**Nodes**				0.001
	Negative	623	-0.082 (-0.518, 0.520)	
	Positive	551	0.061 (-0.348, 0.688)	
**Nodal status**				0.002
	N0	623	-0.082 (-0.518, 0.520)	
	N1	358	0.057 (-0.356, 0.564)	
	N2	127	0.234 (-0.367, 1.008)	
	N3	66	0.055 (-0.264, 0.807)	
**Histology subtype**				1.69E-07

Ductal/NST	889	0.067 (-0.375, 0.682)	
Lobular	92	-0.377 (-0.800, 0.024)	
Medullary	15	-0.024 (-0.303, 0.729)	
Metaplasti	0	NA	
Mixed	138	-0.031 (-0.614, 0.304)	
Mucinous	12	-0.646 (-0.970, -0.451)	
Other	7	0.632 (-0.823, 1.737)	
Tubular/cri	15	-0.288 (-0.692, 0.602)	
Unknown	6		
**Subtype PAM50**			< 2E-16
Basal	113	-0.082 (-0.489, 0.471)	
Claudin-lov	137	0.235 (-0.186, 0.718)	
Her2	108	0.344 (-0.173, 1.045)	
LumA	429	-0.145 (-0.564, 0.242)	
LumB	285	0.226 (-0.342, 1.077)	
NC	4	-0.154 (-0.571, 0.359)	
Normal	98	-0.335 (-0.804, 0.457)	
**3-Gene Classifier Subtype**			< 2E-16
ER-/HER2-	185	0.065 (-0.352, 0.546)	
ER+/HER2-	385	0.080 (-0.400, 0.793)	
ER+/HER2-	395	-0.161 (-0.602, 0.260)	
HER2+	122	0.483 (-0.015, 1.385)	
Unknown	87		
