# Supplementary material for: Hsa-miR-21-3p associates with breast cancer patient survival and targets genes in tumor suppressive pathways
Source: PLoS One. 2021 Nov 19;16(11):e0260327. doi: 10.1371/journal.pone.0260327 (PMC8604322; doi:10.1371/journal.pone.0260327)
Supplement: S4 Table — (PDF) [file pone.0260327.s009.pdf]

Clinical and pathological characteristics of BRCA cohort-2

		miR21-3p mRNA	p-value
	n = 281	median (25 and 75%)	
**Age**			0.965
< 50	64	0.014 (-0.659, 0.584)	
≥ 50	217	-0.009 (-0.554, 0.636)	
**Estrogen receptor**			0.036
Negative	77	0.191 (-0.448, 0.920)	
Positive	197	-0.037 (-0.730, 0.569)	
Unknown	7		
**Progesterone receptor**			0.024
Negative	94	0.194 (-0.456, 0.844)	
Positive	179	-0.092 (-0.781, 0.585)	
Unknown	8		
**ERBB2 status**			0.019
Negative	175	-0.080 (-0.786, 0.538)	
Positive	46	0.469 (-0.329, 1.317)	
Unknown	60		
**HER2 status**			0.003
0 (0 + 1)	217	-0.022 (-0.759, 0.551)	
1 (2 + 3)	51	0.398 (-0.341, 1.195)	
NA	13		
**HER2 combo (ERBB2 status and HER2)**			0.004
0	222	-0.024 (-0.721, 0.552)	
1	53	0.371 (-0.349, 1.171)	
NA	6		
**Tumor size**			0.078
≤ 20	83	-0.104 (-0.782, 0.546)	
> 20	196	0.016 (-0.528, 0.672)	
Unknown	2		
**Histologic Grade**			0.026
1	31	-0.080 (-0.841, 0.365)	
2	128	-0.083 (-0.737, 0.647)	
3	111	0.208 (-0.526, 0.744)	
Unknown	11		
**Nodes**			0.766
Negative	104	0.014 (-0.692, 0.749)	
Positive	146	0.004 (-0.551, 0.612)	
Unknown	31		
**Histology subtype**			0.457
Ductal	234	0.014 (-0.544, 0.712)	
Ductal_lobular	6	0.252 (-0.153, 0.553)	
Ductal_mixed	1	-0.759	
Lobular	29	-0.209 (-1.136, 0.420)	
Lobular_mixed	1	1.26	
Medullary	1	-0.042	
Metaplastic	2	0.788 (0.596, 0.979)	
Mucinous	5	0.022 (-1.454, 0.176)	
Sarcoma	1	(-0.328)	

Tubular	1	(-1.254)
