# Supplementary material for: Hsa-miR-21-3p associates with breast cancer patient survival and targets genes in tumor suppressive pathways
Source: PLoS One. 2021 Nov 19;16(11):e0260327. doi: 10.1371/journal.pone.0260327 (PMC8604322; doi:10.1371/journal.pone.0260327)
Supplement: S5 Table — (PDF) [file pone.0260327.s010.pdf]

Clinical and pathological characteristics of BRCA-TCGA cohort

		miR21-3p mRNA	p-value
	n = 946	median (25 and 75%)	
**Age**			0.053
< 50	270	0.050 (-0.366, 0.0.533)	
≥ 50	676	-0.019 (-0.651, 0.504)	
**Estrogen receptor**			5.07E-05
Negative	216	0.227 (-0.273, 0.636)	
Positive	682	-0.075 (-0.682, 0.432)	
Unknown	48		
**Progesterone receptor**			8.69E-06
Negative	296	0.196 (-0.370, 0.704)	
Positive	599	-0.104 (-0.671, 0.403)	
Unknown	51		
**HER2 status**			5.82E-04
Negative	470	-0.045 (-0.655, 0.467)	
Positive	131	0.177 (-0.317, 0.877)	
Unknown	345		
**Disease stage**			0.994
Stage I	167	0.010 (-0.626, 0.600)	
Stage II	533	-0.019 (-0.547, 0.512)	
Stage III	216	0.020 (-0.587, 0.482)	
Stage IV	15	0.010 (-0.515, 0.719)	
Stage X	10	0.122 (-0.455, 0.169)	
Unknown	5		
**Tumor stage**			2.70E-02
T1	258	0.008 (-0.546, 0.489)	
T2	537	0.030 (-0.542, 0.553)	
T3	118	-0.238 (-0.850, 0.306)	
T4	30	0.211 (-0.299, 0.712)	
TX	3	0.080 (-0.264, 0.589)	
**Nodal status**			0.195
N0	441	-0.012 (-0.543, 0.540)	
N1	319	-0.008 (-0.575, 0.449)	
N2	106	0.060 (-0.433, 0.574)	
N3	62	-0.144 (-0.924, 0.446)	
NX	18	-0.321 (-0.591, 0.566)	
**Nodes**			0.553
Negative	441	-0.012 (-0.543, 0.540)	
Positive	505	0.005 (-0.603, 0.489)	
**Metastasis**			0.536
Negative	776	-0.008 (-0.563, 0.534)	
Positive	170	0.052 (-0.599, 0.426)	
**Subtype PAM50***			<2e-16
Basal-like	155	0.220 (-0.223, 0.179)	
HER2-enrichec	66	0.429 (-0.107, 0.981)	
Luminal A	451	-0.150 (-0.8.2, 0.293)	
Luminal B	160	0.237 (-0.234, 0.811)	
Normal-like	32	-0.469 (-1.057, 0.143)	
